# Cost-effectiveness of continuous glucose monitoring with FreeStyle Libre^®^ in Brazilian insulin-treated patients with types 1 and 2 diabetes mellitus

**DOI:** 10.1186/s13098-023-01208-5

**Published:** 2023-11-25

**Authors:** Luciana Bahia, Karla F. Mello, Lívia Lovato Pires Lemos, Naiane Lima Costa, Eduardo Mulinari, Domingos A. Malerbi

**Affiliations:** 1 Brazilian Diabetes Society, São Paulo, Brazil; 2 IQVIA, Real World Insights, São Paulo, Brazil

**Keywords:** Type 1 Diabetes mellitus, Type 2 Diabetes mellitus, Glucose monitoring, Cost-effectiveness

## Abstract

**Background:**

Hypoglycemia is a barrier to optimal glucose control in the treatment of both type 1 (T1DM) and type 2 diabetes mellitus (T2DM). Blood glucose monitoring is essential in diabetes management. Inappropriate glucose management is associated with high mortality and morbidity. FreeStyle Libre^®^ (FSL) is a continuous glucose monitoring (CGM) system that provides effective, safe, and convenient glucose monitoring, without routine finger pricking. This study aims to estimate the incremental cost-effectiveness ratio (ICER) of the FSL system in comparison to conventional Self-monitoring of blood glucose (SMBG) in T1DM and T2DM patients that require intensive insulin therapy.

**Methods:**

A decision-tree model was developed to compare the cost-effectiveness ratio between FSL and conventional SMBG from the perspective of the Brazilian Public Healthcare System (SUS). The model captures the cumulative rates of acute complications such as severe hypoglicemia and diabetic ketoacidosis, per-event costs, and quality-adjusted life-years (QALYs) gained over a 1-year time horizon in adult and pediatric patients (≥ 4 years old) with T1DM or T2DM. Inputs from the Brazilian health databases, clinical trials, and real-world data were used in the study.

**Results:**

The results demonstrated that, regarding solely severe hypoglicemia and diabetic ketoacidosis events, T1DM have a QALY difference of 0.276, a cost difference of R$ 7.255, and an ICER of R$ 26,267.69 per QALY gained for CGM with FSL, when compared to conventional SMBG. T2DM results demonstrated equally a QALY difference of 0.184, a cost difference of R$ 7290, and an ICER of R$ 39,692.67 per QALY gained, in favour of CGM with FSL.

**Conclusion:**

Our findings demonstrated that FSL is cost-effective in T1DM and T2DM for acute diabetic complications, from a SUS perspective. CGM with FSL can promote safe, convenient, and cost-effective glucose monitoring, therefore contributing to the improvement of the incidence of complications and quality of life.

## Background

Hypoglycemia is a major side effect of some glucose-lowering therapies, in particular, insulin and the insulin secretagogues [1]. The risk of hypoglycemia is a barrier to the optimal glucose control in the treatment of both type 1 (T1DM) and type 2 diabetes mellitus (T2DM), especially in the context of insulin therapy [2]. In the past years, self-monitoring of blood glucose (SMBG) was the main resource to monitor blood glucose. Nonetheless, SMBG is associated with psychological, economic, and social burdens [3, 4]. The monitoring routine is probably the main reason for that, as it requires several finger punctures per day. The frequent and painful SMBG routine might negatively impact the patient’s quality of life and treatment adherence. Although blood glucose monitoring is essential in diabetes mellitus (DM) management, several patients struggle to follow national recommendations (three or four times a day) [5]. SMBG adherence rate may vary between 13.0 and 79.9% in T2DM individuals from low- and middle-income countries [6]. Without the appropriate glucose management, T1DM and T2DM patients may suffer acute and chronic complications, such as hypoglycemia, diabetic ketoacidosis, cardiovascular, ocular, renal, and neurological impairments. Optimized glycemic control is fundamental to minimizing DM mortality and morbidity [7].

FreeStyle Libre (FSL) is a continuous flash glucose monitoring system that provides effective, safe, and convenient glucose monitoring, without routine finger pricking [8–10]. FSL comprises a small, round, disposable, and water-resistant dwelling sensor applied to the back of the arm and changed at 14-day intervals. FSL records interstitial glucose levels every 15 min, which are fed into an app which plots an Ambulatory Glucose Profile, trends of glucose increase or decrease, and other glucose control metrics such as Time in Range, Glucose Variability, Hipoglycemic Episodes, Estimated HbA1c, etc. The sensor does not require daily calibration and can be used by both T1DM and T2DM [11–15].

Currently, Canada and the United Kingdom reimburse FSL for some conditions. The Canadian Agency for Drugs and Technologies in Health (CADTH) recommends FSL reimbursement for T1DM and T2DM patients requiring multiple daily insulins injections and experiencing recurrent hypoglycemia, despite frequent SMBG and efforts to optimize insulin management [16]. The British National Institute of Health and Care Excellence (NICE) recommends reimbursement of FSL for young and adult T1DM patients and for patients with T2DM who use intensive insulin therapy and experience recurrent or severe hypoglycemia [17–19].

To date, FSL is the only continuous flash glucose monitoring device approved in Brazil. It is indicated for people older than 4 years of age with DM, being a substitute for SMBG. Although Brazilian hypoglycemia rates are one of the highest in the world [7], FSL is far from universal availability in the Brazilian Public Healthcare System (SUS). Results from the Hypoglycemia Assessment Tool (HAT) study found that 91.7% of T1DM and 61.8% of T2DM Brazilian patients had at least one hypoglycemic event during the 4 weeks after the start of the follow-up study. These rates were higher than those reported in the global HAT study (83.0% for T1DM and 46.5% for T2DM) and in the Latin-American studies (87.4% in T1DM and 43.8% in T2DM) [7]. The high incidence rates of hypoglycemia in Brazil reinforce the importance of optimizing glucose monitoring and patient access to new, safe and innovative glucose-monitoring technologies.

Given this context, the present study aims to estimate the incremental cost-effectiveness ratio (ICER) of the FSL system in comparison to SMBG in T1DM and T2DM patients that require intensive insulin therapy.

## Methods

### Overall characteristics of the economic model

An economic model was developed in Microsoft Excel comparing FSL with SMBG—the currently standard of care available in SUS. A decision-tree model was developed from a SUS perspective. Inputs from the Brazilian Health Databases, clinical trials, and real-world data were used in the study. The model captures the cumulative rates of acute clinical events (hypoglycemia and diabetic ketoacidosis), per-event costs, and quality-adjusted life-years (QALYs) gained over a 1-year time horizon in adult and pediatric patients (≥ 4 years old) diagnosed with T1DM or T2DM currently treated with intensive insulin therapy.

The present analysis was carried out from the SUS perspective, evaluating the direct medical costs related to the treatment of patients, such as devices and supplies and hospital care. This 1-year time horizon was chosen considering the 6-month follow-up period of the pivotal studies and the outcomes under analysis. Due to the time horizon adopted, no discount rate was applied according to the Brazilian Ministry of Health guideline for health economic studies [20].

**Fig. 1:**
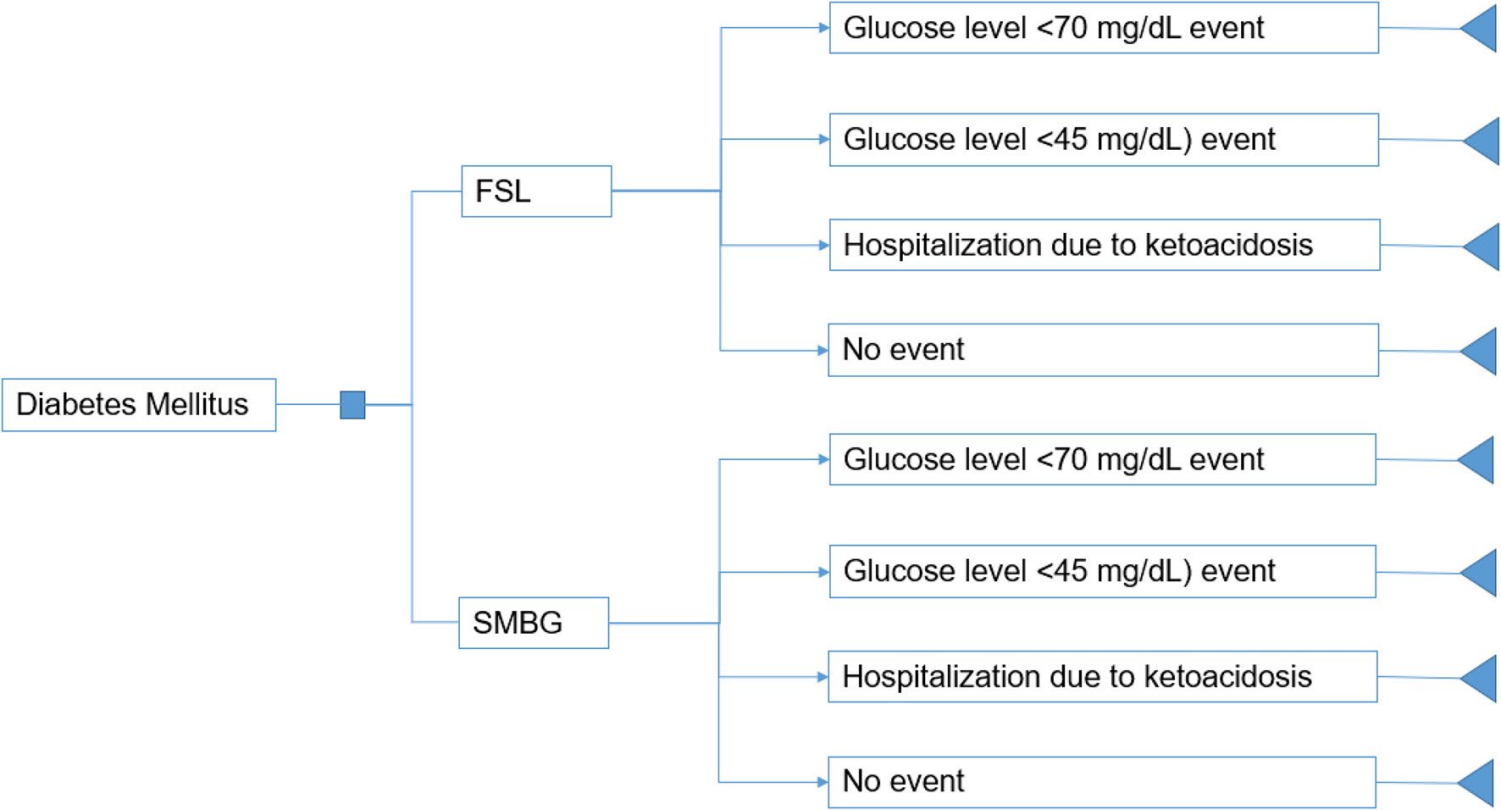
Decision tree used in the model. FSL, FreeStyle Libre; SMBG, Self-monitoring of blood glucose

Two identical decision trees were developed, one for each type of DM, in which patients using FSL or SMBG may experience hypoglycemia or be hospitalized for ketoacidosis or have no events (Fig. 1).

In the analysis we assumed that the average decrease in 24-h hypoglycemia event rates would be constant when evaluated during longer time horizons. Therefore, the relative decrease in the yearly hypoglycemia event rate was applied only once. We also assumed that hypoglycemia and ketoacidosis event rates were constant over the time horizon, and that the results of pivotal studies conducted in patients aged 18 or more years were extensible to younger patients (4–17 years old).

Results are presented as ICER and net monetary benefit (NMB), weighted by the proportion of T1DM and T2DM insulin users in Brazil. ICER is calculated by dividing the difference in total costs by the difference in measures of health outcome (QALY) [21]. NMB is calculated as the benefit of a therapy expressed in monetary terms net of all costs [22]. The effect of uncertainty on ICER was accessed through deterministic and probabilistic sensitivity analyses (PSA). Deterministic sensitivity analyses (DSA) were carried out to assess the impact of model inputs and assumptions on the results, by varying the parameters one at a time to the lower or upper boundary values. Those values were obtained using a 95% confidence interval variation or, if it was unavailable, a 20% variation. PSA evaluated the impact of uncertainty by simultaneously varying cost, utility, and treatment effect parameters as random values within the interval between the lower and upper boundaries of the variables on the results. Parameters varied in DSA and PSA are shown in Tables 1, 2, 3, 4 and 5. All analyses were run with 1000 individuals for 1000 iterations.

**Table 1:** Frequency of self-monitoring of blood glucose in T1DM and T2DM with and without FreeStyle Libre

Parameter	Mean	Lower Bound	Upper Bound	Source
T1DM				
Daily SMBG frequency without FreeStyle Libre	3.5	3	4	Ministry of Health T1DM guideline [5]
Daily SMBG frequency with FreeStyle Libre	0.5	0.37	0.63	Bolinder et al. [24]
T2DM				
Daily SMBG frequency without FreeStyle Libre	3.5	3	4	Ministry of Health T1DM guideline [5]
Daily SMBG frequency with FreeStyle Libre	0.3	0.12	0.48	Haak et al. [25]

T1DM, Type 1 diebetes mellitus; T2DM, type 2 diabetes mellitus; SMGB, Self-monitoring of blood glucose

**Table 2:** Hypoglycemia inputs

Parameter	Mean	Lower Bound	Upper Bound	Source
T1DM				
Non-severe hypoglycemia events per patient-years	99	94.8	103.3	Lamounier et al. [7]
Events of glucose level < 3.9 mmol/L (70 mg/dL) within 24 h	− 25.8%	− 20.6%	− 31.0%	Bolinder et al. [24]
Severe hypoglycemia events per patient-years	9.8	8.5	11.3	Lamounier et al. [7]
Events of glucose level < 2.5 mmol/L (45 mg/dL) within 24 h per patient-years with FSL	5.05	4.38	5.82	Bolinder et al. [24]
Proportion of nocturnal hypoglycemia events	54%	–	–	Lamounier et al. [7]
Proportion of severe hypoglycemia events requiring hospitalization	5.2%	4.2%	6.2%	Lamounier et al. [7]
T2DM				
Non-severe hypoglycemia events per patient-years	25.5	23.7	27.9	Lamounier et al. [7]
Events of glucose level < 3.9 mmol/L (70 mg/dL) within 24 h	− 27.7%	− 22.2%	− 33.2%	Haak et al. [25]
Severe hypoglycemia events per patient-years	6.2	5.2	7.4	Lamounier et al. [7]
Events of glucose level < 2.5 mmol/L (45 mg/dL) within 24 h per patient-years with FSL	3.17	2.66	3.79	Haak et al. [25]
Proportion of nocturnal hypoglycemia events	27%	–	–	Lamounier et al. [7]
Proportion of severe hypoglycemia events requiring hospitalization	3.3%	2.6%	4.0%	Lamounier et al. [7]

Mean numbers were used in base-case analysis. Lower and upper bound values were used in sensitivity analysis

T1DM, Type 1 diebetes mellitus; T2DM, type 2 diabetes mellitus

**Table 3:** Ketoacidosis inputs

Parameter	Mean	Lower bound	Upper bound	Source
T1DM				
Diabetic ketoacidosis hospitalization rates per 100 patient-years without FSL	5.46	3.13	8.31	Roussel et al. [28]
Diabetic ketoacidosis hospitalization rates per 100 patient-years with FreeStyle Libre	2.59	1.85	3.31	Roussel et al. [28]
T2DM				
Diabetic ketoacidosis hospitalization rates per 100 patient-years without FSL	1.7	1.17	2.51	Roussel et al. [28]
Diabetic ketoacidosis hospitalization rates per 100 patient-years with FreeStyle Libre	0.9	0.37	1.23	Roussel et al. [28]

Mean numbers were used in base-case analysis. Lower and upper bound values were used in sensitivity analysis

T1DM, Type 1 diebetes mellitus; T2DM, type 2 diabetes mellitus

**Table 4:** Utility data inputs

Parameter	Mean	Lower bound	Upper bound	Source
Utility related to FSL utilization	0.030	0.024	0.036	Matza et al. [31]
T1DM				
T1DM utility	0.801	0.787	1	Bahia et al. [29]
Disutility of non-severe hypoglycemia events	0.131	0.129	0.133	Lamounier et al. [7] and Laurisden et al. [30]
Disutility of severe daytime hypoglycemic events	0.047	0.033	0.062	Evans et al. [32]
Disutility of severe nocturnal hypoglycemic events	0.051	0.037	0.065	Evans et al. [32]
Disutility of diabetic ketoacidosis events	0.0091	0	0.0287	Peasgood et al. [33]
T2DM				
T2DM utility	0.801	0.787	1	Bahia et al. [29]
Disutility of non-severe hypoglycemia events	0.079	0.077	0.082	Lamounier et al. [7] and Laurisden et al. [30]
Disutility of severe daytime hypoglycemic events	0.047	0.033	0.062	Evans et al. [32]
Disutility of severe nocturnal hypoglycemic events	0.051	0.037	0.065	Evans et al. [32]
Disutility of diabetic ketoacidosis events	0.0091	0	0.0287	Peasgood et al. [33]

Mean numbers were used in base-case analysis. Lower and upper bound values were used in sensitivity analysis

T1DM, Type 1 diabetes mellitus; T2DM, type 2 diabetes mellitus

**Table 5:** Cost inputs

Parameter	Mean	Lower bound	Upper bound	Source
FreeSyle Libre^®^ reader (one-off cost)	R$ 289.90	R$ 260.91	R$ 318.89	Abbott’s suggested price
FreeSyle Libre^®^ sensor (unitary cost)	R$ 289.90	R$ 260.91	R$ 318.89	Abbott’s suggested price
Glucometer (one-off cost)	R$ 70.12	R$ 63.11	R$ 77.13	BPS [35]
Strips (unitary cost)	R$ 0.32	R$ 0.28	R$ 0.35	BPS [36]
Lancets (unitary cost)	R$ 0.13	R$ 0.12	R$ 0.14	BPS [37]
Hospitalization due to severe hypoglycemia	R$ 368.80	R$ 232.12	R$ 429.26	DATASUS [38]
Hospitalization due to ketoacidosis	R$ 555.20	R$ 384.80	R$ 2160.80	DATASUS [38]

Mean numbers were used in base-case analysis. Lower and upper bound values were used in sensitivity analysis

BPS, Banco de Preços em Saúde; DATASUS, Departamento de Informática do Sistema Único de Saúde

## Model inputs

### Use of self-monitoring of blood glucose

The Brazilian Ministry of Health T1DM guidelines recommends SMBG three or four times a day (mean 3.5; lower bound 3; upper bound 4) [23]. Although SMBG is recommended for T2DM patients using insulin, there is no recommended number of measurements per day [5]. For this study, we assume the same recommendations for T1DM and T2DM.

The use of SMBG in patients using FSL may occur when symptoms do not match flash glucose monitoring system readings. As such the number of daily SMBG with FSL for T1DM was assumed to be 0.5 according to the study by Bolinder et al. [24] (lower bound 0.37, upper bound 0.63) and for T2DM was assumed to be 0.3 according to the study by Haak et al. [25] (lower bound 0.12, upper bound 0.48).

### Hypoglycemia events

To consider the potential effect of FSL in the occurrence of severe hypoglycemia, we applied the changes from baseline events of glucose levels < 2.5 mmol/L (45 mg/dL) within 24 h for FSL from the pivotal studies to the incidence of severe hypoglycemia captured in the HAT study with Brazilian T1DM and T2DM patients [7]. Accordingly, the same approach was undertaken to events of glucose levels < 3.9 mmol/L (70 mg/dL) within 24 h, in which the differences observed from baseline with FSL in the pivotal studies were applied to the incidence of non-severe hypoglycemia events captured in the HAT study [7] (Table 2). This approach was also used by other authors [26], and is consistent with the relationship between the occurrence of biochemical hypoglycemia and the occurrence of severe hypoglycemia [27]. Beck et al. [29] found that the risk of severe hypoglycemia in a 3-month period was higher when there was at least one identified biochemical hypoglycemia event (< 70.0 mg/dL or < 5.4 mg/dL) in the Diabetes Control and Complications Trial (DCCT) data set [27].

The HAT study was an observational study developed to explore the hypoglycemia incidence and awareness among insulin treated patients in Brazil. It included 321 T1DM and 293 T2DM, with median time of insulin use of 14.0 and 6.0 years, respectively. The study captured the incidence of severe and non-severe hypoglycemia, the frequency of nocturnal events, and that of hospitalization-requiring events [7].

### Ketoacidosis

The effect of FSL in the incidence of hospitalizations due to ketoacidosis was evaluated in a French study. Roussel et al. [28] used a nationwide database of reimbursement claims to estimate ketoacidosis rates (ICD-10 codes E10.1) in the year before the initiation of FSL and in the first year of the device use. The yearly ketoacidosis rates were reduced by 52% and by 47% after FSL initiation for T1DM and T2DM, respectively [28] (Table 3).

### Utility

Baseline utilities for T1DM and T2DM were extracted from the study by Bahia et al. [29] and considered equal at baseline. This multicenter study evaluated the quality of life and calculated the utility values associated with hypoglycemia in patients with T1DM treated in the SUS [29]. The disutility for severe and non-severe hypoglycemia events were used for glucose level < 2.5 mmol/L (45 mg/dL) events and glucose level < 3.9 mmol/L (70 mg/dL) events, respectively. A regression equation proposed by Laurisden et al. [30] was used to adjust the disutility of hypoglycemic events, lowering the utility as frequency increases (Table 4).

Matza et al. [31] estimated the utility related to FSL use in comparison to SMBG. They interviewed 209 individuals from Edinburgh and London (United Kingdom) using the time trade-off method. The difference of 0.030 between the utilities given to both health states, FSL and SMBG, was applied to the model as the utility associated with FSL use for both T1DM and T2DM [31] (Table 4).

### Costs

The costs associated with SMBG included the provision of a glucometer (one-off cost), disposable lancets, and reagent strips. The costs of these resources were extracted from the SUS public health price panel (BPS—*Banco de Preços em Saúde*), by calculating the weighted average public purchases carried out for the period of competence from January to December 2022. The costs of FSL comprised one reader (1st purchase, one-off cost), and the sensors, replaced at 14-day periods.

The median cost of hospitalization for ketoacidosis was derived from the SUS Hospitalization Information System (SIH/SUS). Public data was extracted from SUS Department of Informatics (DATASUS—*Departamento de Informática do Sistema Único de Saúde*) for the period of competence from January to December 2022, using ketoacidosis ICD-10 codes (E10.1; E13.1 or E14.1) and the reimbursement claims codes from SUS Procedures, Medicines, Orthotics, Prostheses and Special Materials Management System (SIGTAP) for treatment of metabolic disorders (03.03.03.004-6); or treatment of diabetes mellitus (03.03.03.003-8); or pediatric intensive care unit (ICU) level I (day cost; 08.02.01.014-8); or pediatric ICU level II (day cost, 08.02.01.015-6); or pediatric ICU level III (day cost; 08.02.01.007-5); or adult ICU level I (day cost, 08.02.01.010-5); or adult ICU level II (day cost, 08.02.01.008-3); or adult ICU level III (day cost, 08.02.01.009-1) (Table 5). The higher the level of the ICU, the higher the medical resources used and the higher the costs.

The median cost of hospitalization due severe hypoglycemia was derived from SIH/SUS using hypoglycemia ICD-10 codes (E16.0; E16.1; or E16.2) and SIGTAP claim codes. In the HAT study, 5.2% of T1DM and 3.3% of T2DM patients reported hospitalization due to hypoglycemia. These figures were used to weight the cost of severe hypoglycemia [7] (Table 5). The widely accepted definition of severe hypoglycemia in the guidelines refers to episodes of hypoglycemia that require assistance from another person [5, 34]. In this sense and considering that this action does not imply costs from the SUS perspective, only hospitalization costs were considered in the analysis. This assumption lead to an underestimation of the incidence of sever hypoglycemia events that often do not reach the hospital We also assumed that non-severe hypoglycemia events incurred with zero cost to the SUS.

## Results

Over a 1-year horizon, treatment with FSL combined with sporadic SMBG presented a positive outlook for T1DM and T2DM, compared with SMBG alone. The T1DM scenario showed a QALY difference of 0.276, a R$ 7.255 cost difference, and an ICER of R$ 26,267.69 per QALY gained. In comparison, the T2DM results demonstrated a QALY difference of 0.184, a R$ 7290 cost difference, and an ICER of R$ 39,692.67 per QALY gained. The complete results are available in Table 6.

**Table 6:** Cost-effectiveness of FSL compared with SMBG in T1DM and T2DM

Alternative	Total cost	Total QALYs	Cost difference	QALY difference	ICER	NMB
T1DM						
FSL	R$ 8116	0.464	R$ 7255	0.276	R$ 26,268	R$ 3793
SMBG	R$ 861	0.187				
T2DM						
FSL	R$ 8116	0.607	R$ 7290	0.184	R$ 39,693	R$ 56
SMBG	R$ 726	0.423				

T1DM, Type 1 diabetes mellitus; T2DM, type 2 diabetes mellitus; FSL, FreeStyle Libre; SMBG, self-monitoring of blood glucose; ICER, Incremental cost-effectiveness threshold ratio; NMB, Net Monetary Benefit

**Fig. 2:**
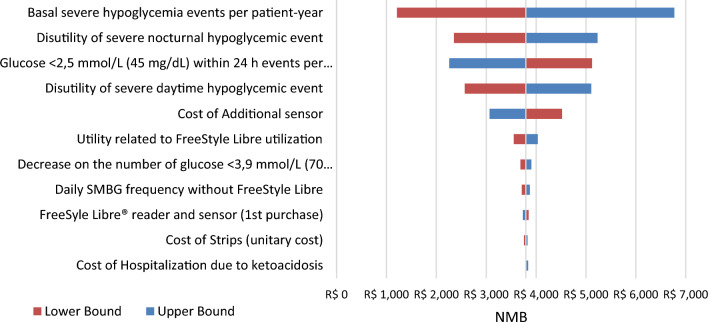
Deterministic sensitivity analysis of NMB for FSL versus SMBG in T1DM. *Note*: The tornado chart displays the 10 parameters with the greatest variation in the DSA. FSL, FreeStyle Libre, NMB, Net Monetary Benefit; SMBG, self-monitoring of blood glucose; T1DM, Type 1 diabetes mellitus

**Fig. 3:**
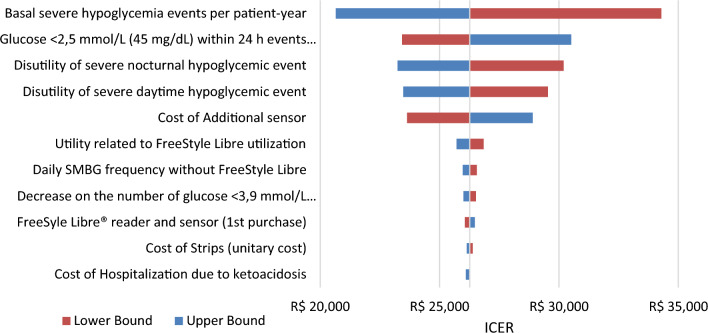
Deterministic sensitivity analysis of ICER for FSL versus SMBG in T1DM. *Note*: The tornado chart displays the 10 parameters with the greatest variation in the DSA. FSL, FreeStyle Libre, ICER, Incremental Cost-Effectiveness Ratio; SMBG, self-monitoring of blood glucose; T1DM, Type 1 diabetes mellitus

**Fig. 4:**
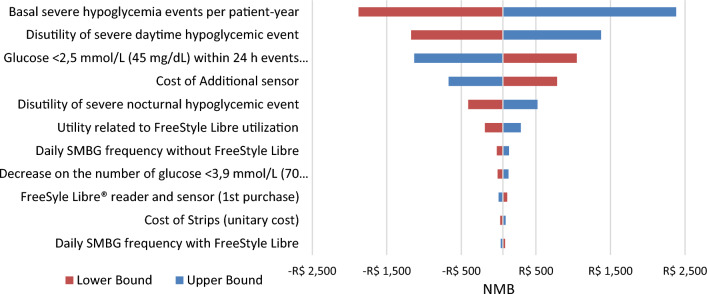
Deterministic sensitivity analysis of NMB for FSL versus SMBG in T2DM. *Note*: The tornado chart displays the 10 parameters with the greatest variation in the DSA. FSL, FreeStyle Libre, NMB, Net Monetary Benefit; SMBG, self-monitoring of blood glucose; T2DM, Type 2 diabetes mellitus

**Fig. 5:**
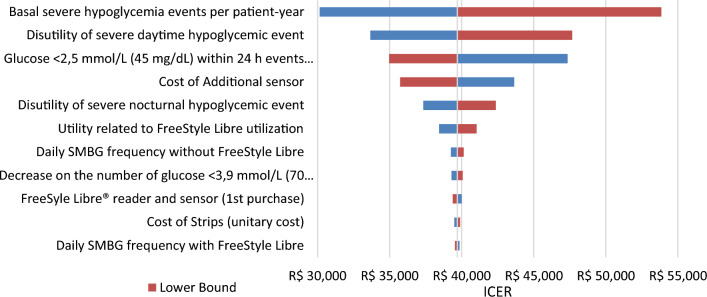
Deterministic sensitivity analysis of ICER for FSL versus SMBG in T2DM. *Note*: The tornado chart displays the 10 parameters with the greatest variation in the DSA. FSL, FreeStyle Libre, ICER, Incremental Cost-Effectiveness Ratio; SMBG, self-monitoring of blood glucose; T2DM, Type 2 diabetes mellitus

As for DSA, basal event-rates of severe hypoglycemia per patient-years, disutility of severe daytime hypoglycemic events and events of glucose < 2.5 mmol/L (45 mg/dL) within 24h per patient-years with FreeStyle Libre were the most sensitive parameters to impact results in both T1DM (Figs. 2, 3) and T2DM populations (Figs. 4, 5).

**Fig. 6:**
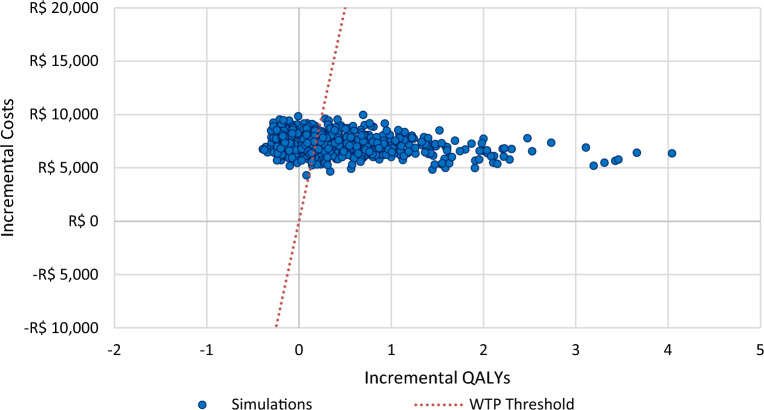
Probabilistic sensitivity analysis scatter plot of FSL-plus-SMBG versus SMBG-only in T1DM. QALY, Quality-Adjusted Life-Years; WTP, Willingness-To-Pay

**Fig. 7:**
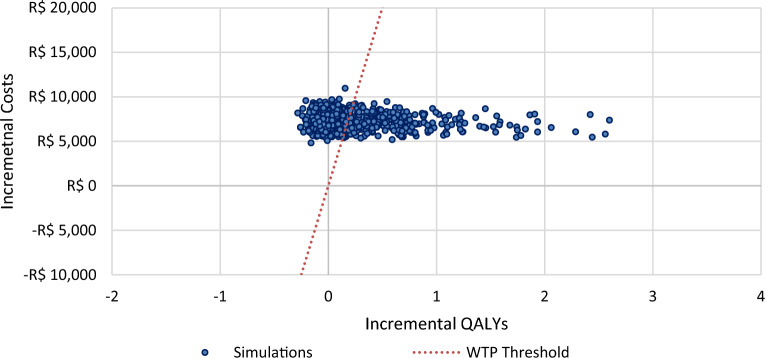
Probabilistic sensitivity analysis scatter plot of FSL-plus-SMBG versus SMBG-only in T2DM. QALY, Quality-Adjusted Life-Years; WTP, Willingness-To-Pay

In 1000 simulations PSA-wise analysis, 64.4% and 58% of the points resulting from T1DM and T2DM, respectively, remain above the willingness-to-pay threshold line of R$ 40,000/QALY in the scatter plot. However, 35.6% of the points from the T1DM and 42% from T2DM analyses remain below thew willingness-to-pay threshold line (Figs. 6, 7).

**Fig. 8:**
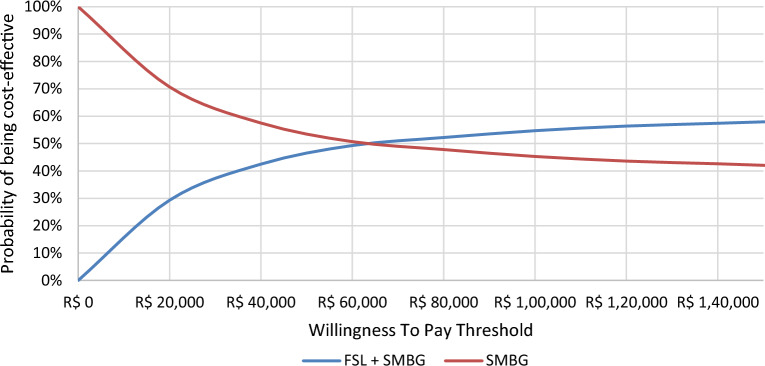
Cost-effectiveness acceptability curve for FSL-plus-SMBG versus SMBG-only in T1DM. FSL, FreeStyle Libre; SMBG, self-monitoring of blood glucose

**Fig. 9:**
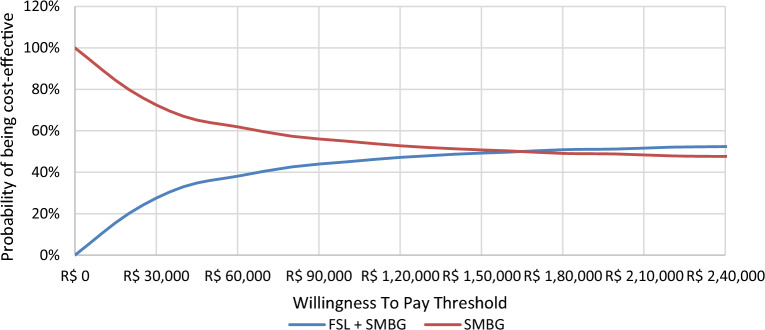
Cost-effectiveness acceptability curve for FSL-plus-SMBG versus SMBG-only in T2DM. FSL, FreeStyle Libre; SMBG, self-monitoring of blood glucose

The cost-effectiveness acceptability curves for T1DM and T2DM are presented in Figs. 8 and 9.

## Discussion

Hypoglycemia affects glycemic control and safety during insulin treatment of both T1DM and T2DM populations [7]. Hypoglycemia is associated with increased morbidity and mortality [7, 39]. Its manifestations within different glucose ranges vary from asymptomatic to severe symptoms of neurological and cardiovascular dysfunction, such as dizziness, confusion, weakness, anxiety, fear, disrupted sleep, cardiac arrythmias and ischemia, convulsions, cognitive disorders, coma and even death [7, 39]. Hypoglycemia has a significant burden on healthcare resources, due to the direct costs of its treatment and the indirect costs of lost productivity, in addition to a great impact on the quality of life of patients and their families [1]. Lamounier et al. [7] estimated that hypoglycemic episodes represented an yearly cost to SUS of R$709 per T1DM patient [range R$0–R$12,364 (mean direct costs: R$640; mean indirect costs: R$69)] and of R$396 per T2DM patient [range R$0–R$10,431 (mean direct costs: R$390; mean indirect costs: R$6)]. Hospitalizations was the main cost driver [7, 40].

Even though pharmacotherapeutic advances have increased in recent years, a significant proportion of patients are still unable to control their blood glucose levels within the recommended limits [39]. Current monitoring based on SMBG has significant limitations, especially regarding low patient adherence to the recommendations established by national and international scientific societies [39].

The FreeStyle Libre system enables patients to measure their glucose levels in the interstitial fluid without routinely pricking their fingers. Also, it reduces the number of steps required to monitor glucose levels, compared to the traditional SMBG approach. This new technology is associated with several outcome benefits. A meta-analysis conducted by Gordon et al. [41] demonstrated a mean HbA1c reduction in T1DM patients of 4.5 mmol/mol (2.6%) [95% CI 3.3–5.6 mmol/mol (2.5–2.7%)] [41]. Dicembrini et al. [42] also reported that FSL is related to an improvement in quality of life and a lower incidence of hypoglycemic events in T2DM patients.

Optimized glycemic control is fundamental to minimizing morbidity. Poor glycemic control in diabetes is associated with serious complications such as sight-threatening retinopathy and renal failure. The most expensive aspect of treating severe hypoglycemia is hospital admission and inpatient care [1]. Oyagüez et al. [39] developed a model to estimate the annual savings with FSL compared with SMBG in the Spanish health system. The results indicate that FSL utilization would reduce 43.1% of the total annual cost per patient. Reduction of severe hypoglycemia was the main contributing parameter for the estimated cost savings [39]. Cost savings were equally observed in the United Kingdom; an annual saving of £234.28 per T1DM patient was reported with FSL use [26]. These data must probably have been of paramount importance to the recent recommendation from the United Kingdom National Health System of continuous glucose monitoring for all T1DM patients [43].

Our analysis was conducted from a SUS perspective. For T1DM patients, it is estimated an ICER of R$ 26,267.69 per QALY. For the T2DM scenario, the ICER was estimated at R$

39,692.67 per QALY. In 2022, the National Committee for Technology Implementation in the Public Health System (CONITEC) established an ICER threshold for reimbursement decisions [44]. The guideline defines that the standard value for most diseases should not overpass one Gross Domestic Product per capita/QALY (R$ 40,000.00/QALY). In other scenarios involving ultrarare diseases or advanced technologies, ICER value can overtake up to three times the reference value [44]. Therefore, both T1DM (R$ 26,267.69) and T2DM (R$ 39,692.67) ICER values would be under the threshold defined by CONITEC.

The impact of uncertainty on ICER was assessed using DSA and PSA. The DSA results were presented in the form of a “tornado chart”, with parameters ordered based on to the extent of the uncertainty variation [45]. The analysis revealed that the ICER was primarily influenced by two key variables: the basal events of severe hypoglycemia per patient-year and disutility of severe nocturnal hypoglycemic event. While both parameters exhibited a larger degree of uncertainty in comparison to the others, it is noteworthy that these critical variables were derived from national epidemiological studies, thereby enhancing the robustness of the dataset. Furthermore, the variation of parameters related to ketoacidosis were not among the most influential in the ICER (data not shown in the manuscript).

Implementation particularities for FSL may exist. Two elements seem to be operational in this regard: one is the sensor reader, a one-off cost item. However, many users do not buy the FSL reader today. The company has developed a mobile application (LibreView^®^) that enables users to read the sensor through cell phones with “Near Field Communication” (NFC) capacity. The NFC technology enables communication between two electronic devices, allowing such features as identification of documents, contactless payment, and scanning FSL sensors. Although NFC is a relatively recent set of communication in mobile phones, there are nowadays more than two billion NFC-enabled devices worldwide. In the long run, NFC technology is expected to be available in most cell phone devices [46]. Therefore, this one-off, significant cost may not be necessary for several patients, which might bring a reduction in the overall cost. The other element is the sensor itself, which must be replaced at 14-day periods. This may bring forth concerns about the system-related annual cost. Nonetheless, the reduction in hypoglycemic episodes and in the need for SMBG supplies with FSL use results in lesser consumption of healthcare resources [25]. Therefore, the overall cost of FSL system might translate into a better control of the disease and a reduction in the consumption of other supplies.

There are some limitations to our model. The first one is related to the ketoacidosis rate, which was based on the French data and may not reflect the Brazilian incidence. The second is related to the time horizon of the analysis, which was of 1 year, despite DM being a chronic disease. We chose to estimate the cost-effectiveness of glucose monitoring with FSL over a 1-year time horizon based on the results of FSL clinical trials in T1DM and T2DM groups. The third limitation is associated with subgroup analyses: results obtained from adult participants were extended to children and adolescents. Therefore, the data might not entirely reflect the other subgroup scenarios.

## Conclusions

The current study pioneers the development of an economic model to estimate the implementation of FSL for T1DM and T2DM patients from the SUS perspective. The results demonstrated that ICER values for T1DM and T2DM patients are under the threshold defined by the Brazilian Committee for Technology Implementation in 2022. FSL's innovative technology can promote safe, convenient, and cost-effective glucose monitoring and therefore contributing to the improvement of the incidence of complications and quality of life for the millions of people living with diabetes in Brazil, with many thousands of children and adolescents among them.

## Data Availability

Not applicable.

## Abbreviations

BPS: SUS Public Health Price Panel (*Banco de Preços em Saúde*)
CADTH: The Canadian Agency for Drugs and Technologies in Health
CONITEC: Brazilian National Committee for Technology Implementation in the Public Health System
DATASUS: SUS Department of Informatics (*Departamento de Informática do Sistema Único de Saúde*)
DCCT: Diabetes Control and Complications Trial
DM: Diabetes Mellitus
DSA: Deterministic sensitivity analyses
FSL: FreeStyle Libre
HAT: Hypoglycemia Assessment Tool
ICER: Incremental Cost-Effectiveness Ratio
ICU: Intensive Care Unit
NFC: Near Field Communication
NICE: British National Institute of Health and Care Excellence
NMB: Net Monetary Benefit
PSA: Probabilistic Sensitivity Analyses
QALY: Quality-Adjusted Life-Years
SIGTAP: SUS Procedures, Medicines, Orthotics, Prostheses and Special Materials Management System (*Sistema de Gerenciamento da Tabela de Procedimentos, Medicamentos e OPM do SUS*)
SIH/SUS: SUS Hospitalization Information System (*Sistema de Informação Hospital*)
SMBG: Self-Monitoring of Blood Glucose
SUS: Brazilian Public Healthcare System (*Sistema Único de Saúde*)
T1DM: Type 1 Diabetes mellitus
T2DM: Type 2 Diabetes mellitus
WTP: Willingness-To-Pay
